# Detection of non-tuberculous mycobacteria and other bacterial pathogens in dental unit waterlines, 2024, Germany: a microbiological single-centre study

**DOI:** 10.3205/dgkh000623

**Published:** 2026-02-09

**Authors:** Lea-Elisa Heinz, Matthias Hannig, Stefan Rupf, Madline P. Gund, Barbara C. Gärtner, Sören L. Becker, Uwe Schlotthauer

**Affiliations:** 1 Institute of Medical Microbiology and Hygiene, Saarland University, Homburg, Germany; 2 Postgraduate Training for Applied Epidemiology (PAE), Department for Infectious Disease Epidemiology, Robert Koch Institute, Berlin, Germany; 3 ECDC Fellowship Programme, Field Epidemiology path (EPIET), European Centre for Disease Prevention and Control (ECDC), Stockholm, Sweden; 4 Clinic of Operative Dentistry, Periodontology and Preventive Dentistry, Saarland University, Homburg, Germany

**Keywords:** non-tuberculous mycobacteria, dental unit waterlines, M. chimaera, M. chelonae, M. gordonae, M. interjectum, M. mucogenicum, P. aeruginosa, Legionella spp., biofilms

## Abstract

**Background:**

Dental unit waterlines (DUWL) might pose infection risks due to the presence of biofilms. A global outbreak of *Mycobacterium (M.) chimaera* through water-carrying medical devices in cardiac surgery highlighted the importance of water for pathogen transmission. We aimed to assess the presence of non-tuberculous mycobacteria (NTM), *Legionella* spp., and *Pseudomonas (P.)* spp. in DUWLs in one tertiary medical centre in Germany to evaluate their potential role as causative agents of infections linked to dental procedures.

**Methods:**

We conducted a cross-sectional, microbiological single-centre study at Saarland University Medical Centre in Homburg, Germany from May to July 2024. We examined 42 DUWLs once before the daily patient care started. After DUWL flushing, 500 mL water samples were collected. *Legionella* spp. were detected after up to 10 days of incubation, *Pseudomonas (P.)* spp. via membrane filtration and 48-hour incubation, and NTM using liquid and solid cultures with up to eight weeks of incubation. Clinically relevant NTM species such as *M. chimaera* and *M. chelonae* were identified using a GenoType line probe assay. We calculated frequencies and proportions of positive samples.

**Results:**

Bacterial growth was detected in 36 of 42 water samples. A total of 43 NTM species were detected in 35 (83%) water samples. The most common species was *M. chimaera* (n=27), followed by *M. chelonae* (n=7), *M. gordonae* (n=4), *M. interjectum* (n=4), and *M. mucogenicum* (n=1). Multiple NTM species were detected in five samples, including one with four distinct species. One specimen contained only *Legionella* spp., while two of the samples harbored two pathogens each: *P. aeruginosa* with *M. chimaera*, and *Legionella* spp. with *M. chelonae*.

**Conclusion:**

The large proportion of NTM detection highlights the importance of frequent bacterial monitoring in DUWLs for enhanced water quality control, which is not commonly implemented. Further research is needed to address the clinical relevance of NTM in DUWL and to evaluate measures to improve patient safety, such as the use of various disinfectant methods to sustainably reduce NTM concentrations.

## Zusammenfassung

**Hintergrund:**

Wasserleitungen von Dentaleinheiten können aufgrund von Biofilmen ein Infektionsrisiko darstellen. Ein weltweiter Ausbruch von *Mycobacterium (M.) chimaera* durch wasserführende medizinische Geräte in der Herzchirurgie hat die Bedeutung von Wasser für die Übertragung von Krankheitserregern deutlich gemacht. Unser Ziel war es, das Vorkommen von nicht-tuberkulösen Mykobakterien (NTM), *Legionella* spp. und *Pseudomonas (P.)* spp. in Wasserleitungen in Dentaleinheiten in einem tertiären medizinischen Zentrum in Deutschland zu untersuchen, um ihre potenzielle Rolle als Erreger von Infektionen im Zusammenhang mit zahnärztlichen Eingriffen zu bewerten.

**Methoden:**

Wir führten zwischen Mai und Juli 2024 eine mikrobiologische Querschnittsstudie an einem einzelnen Zentrum, dem Universitätsklinikum des Saarlandes in Homburg, durch. Vor Beginn der täglichen Patientenversorgung wurden 42 Wasserleitungen von Dentaleinheiten einmalig untersucht. Nach dem Spülen der Systeme erfolgte die Entnahme von 500 mL Wasserproben. *Legionella* spp. wurden nach bis zu 10 Tagen Inkubation nachgewiesen, *P. aeruginosa* mittels Membranfiltration und 48-stündiger Inkubation und NTM sowohl in Flüssig- als auch Festmedien mit bis zu acht Wochen Inkubationszeit. Klinisch relevante NTM-Arten, wie *M. chimaera* und *M. chelonae*, wurden mittels GenoType-Line-Probe-Assay identifiziert. Zur Auswertung wurden die Häufigkeiten und Anteile positiver Proben berechnet.

**Ergebnisse:**

In 36 von 42 Wasserproben wurde Bakterienwachstum festgestellt. Insgesamt wurden 43 NTM-Arten in 35 (83 %) Wasserproben nachgewiesen. Die häufigste Art war *M. chimaera* (n=27), gefolgt von *M. chelonae* (n=7), *M. gordonae* (n=4), *M. interjectum* (n=4) und *M. mucogenicum* (n=1). In fünf Proben wurden mehrere NTM-Arten nachgewiesen, darunter eine mit vier verschiedenen Arten. Eine Probe enthielt nur *Legionella* spp., während zwei der Proben jeweils zwei Krankheitserreger enthielten: *P. aeruginosa* mit *M. chimaera* und *Legionella* spp. mit *M. chelonae*.

**Schlussfolgerung:**

Der hohe Nachweisanteil an NTM unterstreicht die Bedeutung einer regelmäßigen mikrobiologischen Überwachung von Wasserleitungen in Dentaleinheiten zur Sicherstellung der Wasserqualität, die bislang häufig nicht standardisiert erfolgt. Weitere Studien sind erforderlich, um die klinische Relevanz von NTM in Wasserleitungen von Dentaleinheiten systematisch zu untersuchen und effektive Maßnahmen zur Verbesserung der Patientensicherheit zu evaluieren, wie beispielsweise den gezielten Einsatz unterschiedlicher Desinfektionsverfahren zur nachhaltigen Reduktion der NTM-Konzentrationen.

## Background

Dental treatment units are important to allow for dental procedures in patients. Such units are equipped with dental unit waterlines (DUWL), which can potentially be a risk to patient health during dental treatments [1]. Dental unit waterlines are the pipes inside the dental chair that carry water from external supply systems to the dental clinic or office. DUWLs as well as handpieces may become colonized by bacteria originating from the municipal water supply. Biofilm formation in these systems can lead to the spread of a wide range of microorganisms, including environmental and opportunistic pathogens, e.g. *Legionella* spp., *Pseudomonas* spp., and non-tuberculous mycobacteria (NTM) [2], [3], [4]. The water delivered through these pipes needs to have low levels of microorganisms to ensure it is safe for patients [5]. Dental units are equipped with disinfection systems that use chemical and physical methods to minimize the bacterial load of the water used for patient treatment [6].

Among opportunistic microorganisms, NTM have recently received more attention due to their resistance to common disinfectants and their ability to persist in water systems [7], [8]. NTM represent a broad range of species belonging to the genus Mycobacterium. In contrast to *Mycobacterium (M.) tuberculosis* complex, NTM survive in natural habitats, such as soil and aquatic environments and can be divided into two classes, based on their growth – slow and fast-growing NTM [9]. Typical slow-growing mycobacteria are *M. avium*, *M. gordonae*, *M. intracellulare*, *M. kansasii* and *M. marinum*. Fast-growing mycobacteria, such as* M. abscessus* ssp. *abscessus*, *M. abscessus* ssp. *bolettii*, *M. chelonae, M. fortuitum *and*, M. mucogenicum* already show cultural growth after about seven days [10].

NTM are often non-pathogenic in immunocompetent individuals, but in vulnerable population groups, certain NTM species can give rise to severe infections [11]. DUWL as water-emitting medical devices, represent a potential source of NTM exposure to patient and staff in clinical practice [12]. 

The clinical relevance of such waterborne transmission routes was highlighted by a global outbreak of NTM infections, caused by *M. chimaera*, which was transmitted through to water-carrying medical devices (so-called heater-cooler units) in cardiac surgery. This incident underscored the critical role of water as a vector for pathogen transmission in healthcare settings [13], [14], [15]. Recent studies found that NTM pose a significant health risk in dental practices. Already in 1998, Barbeau et al. [16] reported NTM in biofilms of medical and dental equipment as a potential source of serious hospital-acquired infections. Realpe et al. [2] found that 59% of dental treatment units in Caracas, Venezuela and Quito, Ecuador, were contaminated. Especially immunocompromised patients have been identified as the most affected community for healthcare-acquired NTM infections from healthcare facility water systems [17]. NTM in water environments can be a source of infection, which can lead to serious odontogenic infections. *M. chimaera* has an increased clinical significance in patients with cystic fibrosis or chronic obstructive pulmonary disease (COPD). It may appear that immunosuppressed for other reasons are also at increased risk of infection with *M. chimaera*, but no detailed study data are available on this [15].

Findings highlight the need for better control measures and the enhancement of detection and identification methods for NTM, as a crucial step in preventing infections in healthcare-associated settings [18]. 

Regulatory authorities establish standards and guidelines for the water quality from DUWL as a component of routine infection control. The Commission for Hospital Hygiene and Infection Prevention (KRINKO) is mandated to develop national recommendations for preventing healthcare-associated infections in Germany. The 2006 recommendation, which has not been updated since, included annual microbiological monitoring, particularly focusing on the quantification of *Legionella* spp. and *P. aeruginosa* [19]. In April 2024, the German Working Group for Hygiene in Dentistry (DAHZ) published updated implementation guidelines for hygiene measures in dentistry [20]. Once water is supplied from the drinking water network to a medical device, such as dental treatment units, it is considered process water. Both KRINKO and DAHZ recommended adherence to the microbial limits of the German Drinking Water Ordinance (TrinkwV), although the ordinance does not formally apply to dental units [19], [20], [21]. According to the TrinkwV, colony counts in water systems should not exceed 100 colony forming unit (CFU)/mL) [21]. 

For pathogens such as NTM, there is no routinely implemented testing of the water quality control in Germany, and national data on the relevance of NTM in DUWL are scarce. In this study, we investigated the occurrence of NTM, *Legionella* spp., and* P. aeruginosa* in DUWL in a medical centre in Germany to identify potential microbiological risks associated with dental treatment practices. 

## Methods

### Study design

We conducted a cross-sectional, microbiological single-centre study at the Saarland University Medical Centre, Germany. From May to July 2024, a total of 42 DUWL, which included all dental chairs in the Department of Dentistry, were examined once before the daily patient care started. 

### Sampling

The samples were taken by the hospital hygiene staff from the Institute of Microbiology and Hygiene. All dental units are connected to the municipal drinking water supply. To minimize the influence of stagnant water, the DUWLs were flushed for 30 seconds before the sampling started. 

Per unit, approximately 500 mL of water were collected in sterile containers, prefilled with sodium thiosulfate (20 mg/L, LP Italiana SPA, Milano, Italy) to neutralize residual disinfectants, aware that an oxidative disinfectant is used in the treatment chair system [22]. Mak et al. revealed that sodium thiosulfate did not significantly affect *M. chimaera* recovery [23]. Samples were processed immediately in the microbiological laboratory by trained laboratory technicians. Patient samples were not examined in this study.

### Microbiological analysis of the water samples

CFU were determined by analyzing the number of CFUs in 1 mL at 36±2°C after 48 hours incubation in nutrient-rich and peptone-containing culture media, DEV medium (Xebios Diagnostics Group, Düsseldorf, Germany), following the ordinance for drinking water.

For the detection of *Legionella* spp., we follow the ISO 11731, Picture J.1 Matrix B, procedure 1 [24] and using the BCYE+AB medium (Xebios Diagnostics Group, Düsseldorf, Germany). From each water sample 1 mL was spread on BCYE+AB medium and the samples were incubated at 36°C for 10 days and read on day three, five and seven for bacterial growth. 

*P. aeruginosa* were isolated by filtering 100 mL of water sample through a membrane with a 0.45 µm membrane (Merck KGaA, Darmstadt, Germany). The membrane was incubated, using the CN medium (Xebios Diagnostics Group, Düsseldorf, Germany), at 36°C for 48 hours. Fluorescent colonies were identified as *P. aeruginosa*. (Tab. 1)

### Non-tuberculous mycobacteria in water samples

Liquid culture tubes were utilized for broth-based mycobacterial growth using the Mycobacteria Growth Indicator Tube (BACTEC™ MGIT™ 960 incubator, Becton Dickinson, Heidelberg, Germany). Solid culture was also used to grow mycobacteria (Loewenstein-Jensen and Stonebrink Agar, Becton Dickinson, Heidelberg, Germany). As part of the quality control, all culture media were tested for sterility and their target performance. Samples were incubated for eight weeks. In case of a positive signal in MGIT, the line probe assay GenoType NTM-DR (Hain Lifescience, Nehren, Germany) was used to identify the species- or subspecies-level of major clinically relevant NTM [25], [26]. After DNA extraction, amplification and hybridization were performed according to the assay protocol. Master mixes with amplification mixes A and B were prepared, 5 µL of extracted DNA was added for polymerase chain reaction (PCR). PCR amplification was performed as follows: 1 cycle at 95°C for 15 min, 10 cycles of 30 s at 95°C and 120 s at 65°C, followed by 20 cycles of 25 s at 95°C, 40 s at 50°C, and 40 s at 70°C, with a final extension at 70°C for 8 min. Reverse hybridization and detection were done in a shaking water bath. The developed strips were analyzed in conformance with the manufacturer’s chart [27], [28], [29]. 

### Data analysis

We conducted descriptive analyses, and results are displayed in tables using frequency counts and proportions. We investigated the mean and median, the range (min-max), and the interquartile range (IQR 25^th^; 75^th^ percentile) to summarize the duration for NTM growth in the figure. 

## Results

A total of 43 NTM species were identified in 35 (83%) of 42 water samples (Table 1 (Tab. 1)). The most frequently detected NTM species was *M. chimaera* (n=27), followed by *M. chelonae *(n=7). Other identified NTM species included *M. gordonae* (n=4), *M. interjectum* (n=4), and *M. mucogenicum* (n=1). 

The growth of more than one NTM was detected in a total of five samples. Simultaneous growth of *M. chimaera* and *M. interjectum* was observed in three specimens. One sample showed the presence of three different NTM species (*M. chimaera*, *M. gordonae*, *M. chelonae*), while another showed the growth of four NTM species (*M. chimaera*, *M. interjectum*, *M. chelonae*, *M. mucogenicum*).

**Figure 1:**
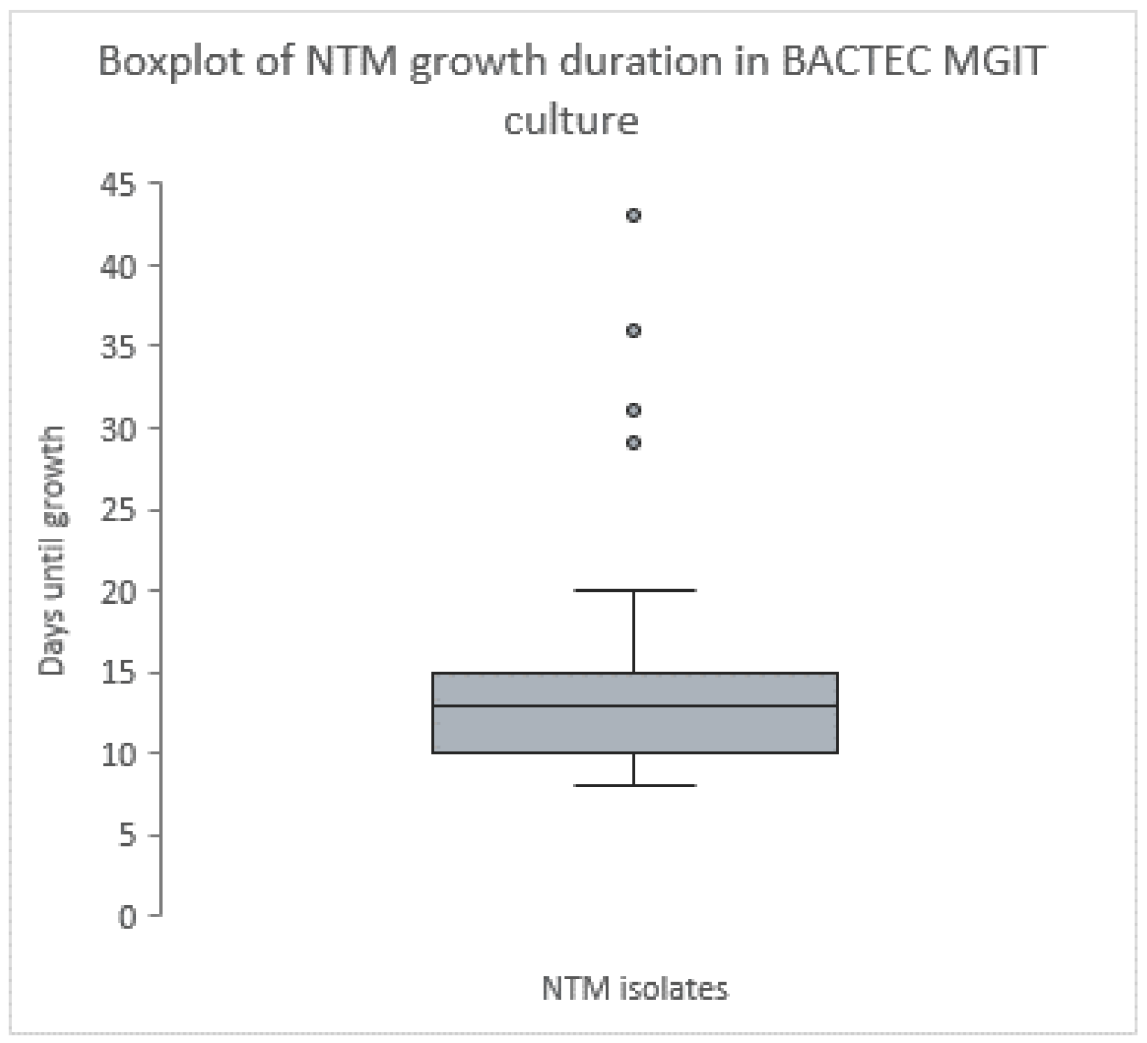
The horizontal line in the boxplot shows the median with 13 days. Values are ranging from the first quartile (Q1=10 days) to the third quartile (Q3=15 days), which is identical to the mean value. The whisker shows the range excluding outliers. The dots represent the outliers, Saarland, Germany, May–July 2024.

Figure 1 (Fig. 1) shows the NTM growth duration in BACTEC MGIT culture across all 35 NTM isolates. The mean duration for NTM growth in BACTEC™ MGIT™ liquid culture was 15 days ranging from eight (*M. chimaera, M. chelonae*) to 43 days (*M. gordonae*). 

Of the 42 water samples tested, only one sample exceeded the acceptable total CFU count at 36±2°C. The permissible total value of 100 CFU/mL was exceeded threefold.

In one sample, one CFU of *P. aeruginosa* was detected. Additionally, *Legionella* spp. were identified in two samples, each containing one CFU. 

Different bacterial species were identified in 36 of the 42 water samples. While one sample contained only *Legionella* spp., two of the samples harbored two distinct pathogen species (n=1, *P. aeruginosa* and *M. chimaera*; n=1, *Legionella* spp. and *M. chelonae*). 

## Discussion

NTM are ubiquitous in the environment, e.g. in water drained surroundings and they can cause serious infections, especially in immunocompromised patients. The large proportion of NTM detected (83%) in DUWLs in this study highlights the need for frequent bacterial monitoring in DUWL water quality controls. The most dominant species of NTM detected in DUWL samples was *M. chimaera* (63%), followed by *M. chelonae* (16%). 

Arvand et al. investigated the presence of *P. aeruginosa* and *Legionella* spp. but not NTM and showed a much higher contamination rate especially for *Legionella* spp. from water of DUWL in dental practices in Hesse, Germany. The study by Arvand et al. evaluated the effects of individual decontamination measures. These included more intensive rinsing, more intensive circulation and stricter adherence to the manufacturer’s recommendations regarding routine cleaning and disinfection measures. The study highlights the risk of exposure for patients and personnel and the need for effective strategies to reduce microbial contamination [30]. 

Different NTM species have been found that have been associated with various health conditions:

- *M. chimaera* was first described in 2004 by a group of scientists in Italy. Initially, it was not regarded as highly clinically relevant [31]. Sax et al. published in 2015 a prolonged outbreak of *M. chimaera* infections after open chest heart surgeries [13]. Outbreak investigations and examinations revealed contaminations of heater-cooler units (HCU) connected to the heart-lung machine during heart surgeries, confirmed by whole genome sequencing. Trudziniski et al. detected *M. chimaera* in water-carrying medical devices, the extracorporeal membrane oxygenation system (ECMO) [32].
- *M. chelonae* is one of the fast-growing NTM, which can be ubiquitous found in water and soil. Commonly it is associated with skin and soft tissue infections. More frequently surgical site infections and catheter related infections occur, especially in immunocompromised patients [33].
- *M. gordonae* is often considered to have a low pathogenicity and is commonly found in the environment. In 2015, Prabaker et al. described a pseudo-outbreak of *M. gordonae* in a newly opened hospital by identifying a reservoir of *M. gordonae* in potable water supply which led to endemic contamination of clinical specimens [34].
- A few cases of *M. interjectum* infections are described in the literature. Mirant-Borde et al. described in 2013 one case of lung infection by a 62-year male patient who had a productive cough, weight loss, and night sweat. Further laboratory investigations identified *M. interjectum* in lung specimens [35]. Yamaguchi et al. described in 2024 the first case of *M. interjectum* pulmonary disease case in Japan [36]. NTM infections with this species are rare and very uncommon.
- *M. mucogenicum* infections are rarely described. In 2012, Ashraf et al. described an outbreak of *M. mucogenicum* bloodstream infections in an outpatient setting [37].

Public drinking water is usually filtered before the entrance of the building. However, due to their limited pore size and permeability, these filters are not able to retain all the microorganisms contained in the water. Microorganisms, which enter the domestic drinking water installation, can multiply under certain conditions, e.g. if the water stagnates, if unsuitable materials are used or if a biofilm grows within the pipes. Even dental treatment units that are only indirectly connected to the drinking water installation still draw their water from this installation and underlie the same microbiological risks. These conditions can represent potential entry points for opportunistic pathogens, such as NTMs, *Legionella* spp. or *P. aeruginosa*. Water is a non-sterile medium and inhabits various microorganisms. Microbiological tests in accordance with the Drinking Water Ordinance are subject to an indicator principle. This means that pathogen groups can be detected quickly and easily using standardized methods. Lengthy testing procedures such as the detection of mycobacteria are not currently established for microbiological parameters in the Drinking Water Ordinance. However, the Drinking Water Ordinance also clearly states that pathogens that can be transmitted through drinking water must not be present in drinking water in concentrations that could cause harm to human health [21]. Our findings demonstrate that a routine microbiological examination of dental unit waterlines, with no deviations in standard microbiological parameters, does not guarantee the absence of NTMs, even when common biofilm-forming pathogens such as *P. aeruginosa* are not detected. Medical devices used with drinking water often provide good environmental conditions for the growth of NTMs, e.g. the temperatures between 30 and 35°C or longer periods of non-use. Although, we did not investigate the transmission from dental unit to the treated patients, there is a risk of transmission during the dental procedures where they can cause colonization or infection. 

This study was conducted as a single-centre investigation. Therefore, the transferability to other facilities may be limited and may depend on factors such as the manufacturer and maintenance status of the dental units as well as the local water supply. In addition, the NTM testing was performed only once on each dental unit. Therefore, dental units that tested negative for NTM in this study may yield positive results in subsequent tests. A negative result in the baseline test is no guarantee that a biofilm will not form in the system over time, which in turn can harbor nutrients for NTMs. Additionally, the potential transmission of NTM to patients via the dental units cannot be specified. 

The specific conditions of dental treatment units, including direct patient contact (partly to open wounds), aerosol generation, and complex water systems, may increase the risk of infections compared to conventional water installations, such as tap water.

Further research on the spread of NTM, e.g. in other water-carrying medical devices, should be carried out. Additionally, studies are warranted to elucidate whether these findings are applicable to other dental clinics or treatment centers. Most importantly, it is unclear whether the presence of NTM in water poses a relevant health risk to the patient or whether it is a largely asymptomatic colonization or infection, if at all. The risk has been demonstrated for *M. chimaera* during the highly artificial situation of infection during an open-heart surgery, but not yet for the other species, especially not using the natural route of drinking water. 

## Limitations

Our study has the limitation that we did not investigate the effectiveness of disinfection measures and disinfection products. Therefore, we do not know whether regular or even intensified disinfection of the systems has a lasting effect on the elimination of NTM in the system. 

## Conclusion

Our study highlights a substantial presence of NTM, particularly *M. chimaera*, in DUWL. These findings suggest that water quality testing protocols should be reviewed, although the actual clinical risk of infection remains to be determined. To minimize the risk for patients, the WHO recommend specific control strategies and define measures in their guidelines for drinking-water quality. Control measures to counter their survival and growth in distribution systems and building water systems include maintaining adequate disinfectant residuals, managing water temperatures, minimizing periods of water stagnation and low flow rates, keeping distribution and building systems, including distal devices (e.g. showerheads) clean and selecting plumbing materials that do not support microbial growth [38]. If it is known that patients suffer from immunosuppression or belong to particularly vulnerable groups, it might be useful to avoid the use of tap water in the treatment unit and prefer sterile alternatives, although this is not explicitly mentioned in current guidelines [39]. Further studies are needed to evaluate the effectiveness of disinfection measures on NTM in water.

## Notes

### Authors’ ORCIDs

- Hannig M: https://orcid.org/0000-0003-0669-6881
- Rupf S: https://orcid.org/0000-0002-1551-9935
- Gund MP: https://orcid.org/0000-0001-9053-8864
- Gaertner BC: https://orcid.org/0000-0002-5234-7634
- Becker SL: https://orcid.org/0000-0003-3634-8802
- Schlotthauer U: https://orcid.org/0009-0006-6793-4192

### Ethical approval 

None.

### Funding

None. 

### Acknowledgments

We thank Indrite Backes, Anja Linz and Sandra Trarbach who collected the samples, the members of our water analysis laboratory (Anika Berndt, Martina Birke, Julian Haupenthal, Veronika Gerber, Sandra Rauch, Monika Schmitt, Teresa Türr, Nina Walzer), as well as the team of our diagnostic mycobacteria laboratory (Maike Decker, Sabine Freis, Lisa Freiwald, Diana Velten). We are also grateful to the entire team of the German Postgraduate Training for Applied Epidemiology (PAE) at the Robert Koch Institute, in particular Ida Sperle-Heupel, for their continuous support and valuable contributions to this work.

### Competing interests

The authors declare that they have no competing interests.

The author Lea-Elisa Heinz was a fellow of the German Postgraduate Training in Applied Epidemiology (PAE) associated with the ECDC Fellowship Programme. The views and opinions expressed herein do not state or reflect those of ECDC. ECDC is not responsible for the data and information collation and analysis and cannot be held liable for conclusions or opinions drawn.

## Figures and Tables

**Table 1:**
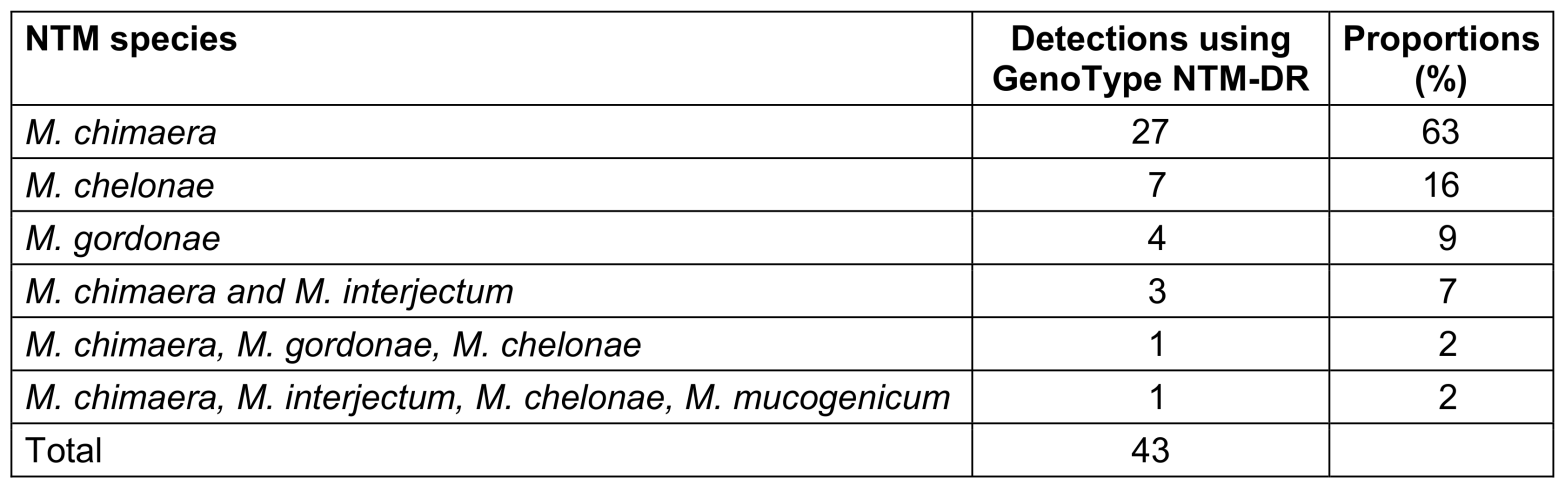
Distribution of NTM species (n=43) detected in 35 water samples testing positive for NTM, Saarland, Germany, May–July 2024.
